# A Comparison Between the Efficacy of Scalpel and Laser Procedures for Treating Gingival Hyperpigmentation: A Case Report

**DOI:** 10.7759/cureus.27954

**Published:** 2022-08-12

**Authors:** Safiya Hassan, Prasad Dhadse, Pavan Bajaj, Chitrika Subhadarsanee

**Affiliations:** 1 Department of Periodontics, Sharad Pawar Dental College, Datta Meghe Institiute of Medical Sciences, Wardha, IND

**Keywords:** physiologic pigmentation, melanin, lasers, electro surgery, cryosurgery, gingiva

## Abstract

Gingival hyperpigmentation is a hereditary feature in populations. Gingival pigmentation not only affects aesthetics but also has a negative psychological effect. For many people, gingival hyperpigmentation is a severe cosmetic problem. Although black gums are not a medical issue, many individuals find them unsightly. The pigmentation of gingiva contributes to the harmony of the smile in a significant way. A periodontal plastic surgery procedure called gingival depigmentation eliminates or reduces hyperpigmentation. Different treatment approaches for gingival depigmentation have been documented, such as scalpel, electrosurgery, diamond burs, chemical methods, cryosurgery, and lasers. According to studies, cryosurgery and lasers are the best procedures since they provide better aesthetic outcomes and low recurrence rates. The coordination of treatment plans and the choice technique are influenced by the patient's skin tone, the degree of gingival pigmentation, the lip line, the upper lip curvature, aesthetic concern, and treatment expectations. This case report, which involves a 23-year-old female patient, provides a comparison between the efficacy of scalpel and laser procedures for treating gingival hyperpigmentation. The patient's left side received diode laser treatment, while the right received scalpel treatment. Scalpel depigmentation caused the treated area to recover without incident. The benefits of laser depigmentation include a bloodless surgical field and recovery without complications. No postoperative pain, bleeding, infection, or scars were seen on the first and consequent visits. The recovery went smoothly. The patient was satisfied with the treatment modality, and the outcomes were outstanding, according to the patient. There was no re-pigmentation throughout the follow-up period. This split-mouth study will provide information regarding soft tissue healing using two different approaches in the same patient.

## Introduction

The beauty of a smile is influenced by the gingival tissues' state of health and appearance. The gingival color is influenced by the quantity and size of the underlying blood vessels, the thickness of the epithelium, the level of keratinization, and the pigments that are naturally present inside the gingiva [1]. The primary pigments in the oral mucosa include carotene, melanin, hemoglobin, and oxyhemoglobin [2]. A brown pigment called melanin is produced by melanocytes in their cytoplasm. Chemical messengers such as melanocyte-stimulating hormones are created once melanocytes are activated by factors such as stress hormones and sunlight.

Stimulated melanocytes then produce melanin-rich granules known as melanosomes. Tyrosine is converted into the molecule dihydroxyphenylalanine (DOPA) by the enzyme tyrosinase. Additionally, DOPA is converted by tyrosinase into the secondary metabolite dopaquinone. A series of steps converts dopaquinone into either dark melanin (eumelanin) or light melanin (pheomelanin). Melanin is transported to skin and oral epithelial keratinocyte cells [3]. The pigmentation of the areas of the gums is attributed to excessive melanin production in the basal and supra-basal layers of cells of the gingival epithelium [4]. Gingival hyperpigmentation is a physiological abnormality that occurs mainly in people with dark skin. Periodontal plastic surgery, called gingival depigmentation, uses several surgical techniques to eliminate or lessen hyperpigmentation [5]. The patient's desire for a better look is the primary and most significant justification for performing depigmentation. Depigmentation treatments of many kinds have been used. The choice of technique should be based on clinical knowledge and personal preferences. The surgical removal of unwanted pigmentation using scalpels was one of the first and most common treatments [6]. There is very little information in the literature about surgical depigmentation. The technique involves surgically eliminating the gingival epithelium and a thin connective tissue layer beneath it. The stripped connective tissue is then healed using secondary intention. The newly formed epithelium is free of melanin color [6]. This article compares the efficacy of scalpel and laser procedures for treating gingival hyperpigmentation.

## Case presentation

**Figure 1:**
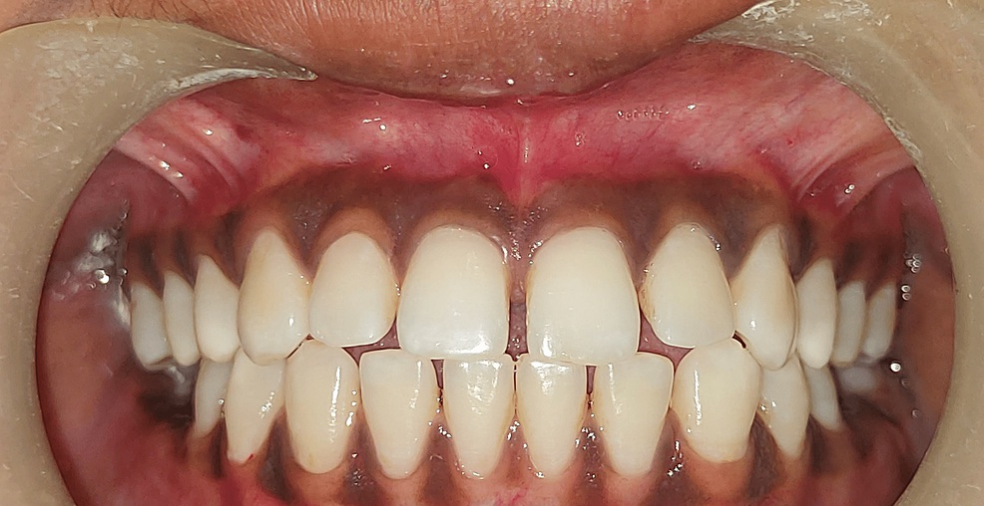
Baseline view The image shows gingival hyperpigmentation in the upper anterior region (aesthetic zone)

A 23-year-old female patient presented with the complaint of black gums to the Department of Periodontology at the Sharad Pawar Dental College, Sawangi Meghe, Wardha. It had been present from childhood, which suggested physiological melanin pigmentation. Clinical examination indicated considerable gingival melanin pigmentation in the upper and lower jaws. The patient was a non-smoker with a wheatish complexion. There was no family history of gingival pigmentation. The patient was in good health overall and had good oral hygiene. The patient was aware of the numerous treatment choices and the possibility of re-pigmentation after a specific period. Phase I treatment was performed on the first appointment. A split-mouth approach was planned, contrasting scalpel and laser techniques. Figure 1 shows the baseline view of hyperpigmented areas.

**Figure 2:**
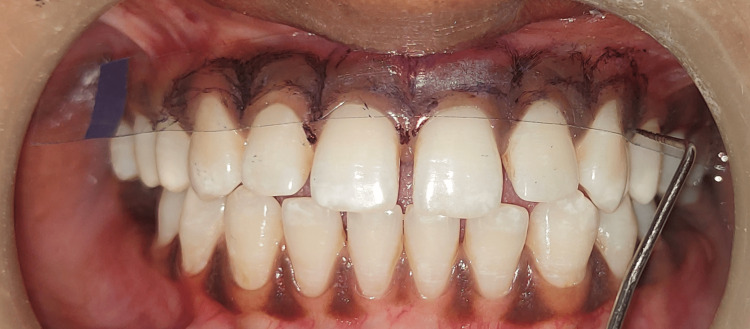
Figure depicting the hyperpigmented area The image shows the marking of the hyperpigmented area using a template in the anterior aesthetic zone

Lignocaine infusion with adrenaline was used locally as a method of infiltration. Figure 2 depicts the extensions of the hyperpigmented site.

**Figure 3:**
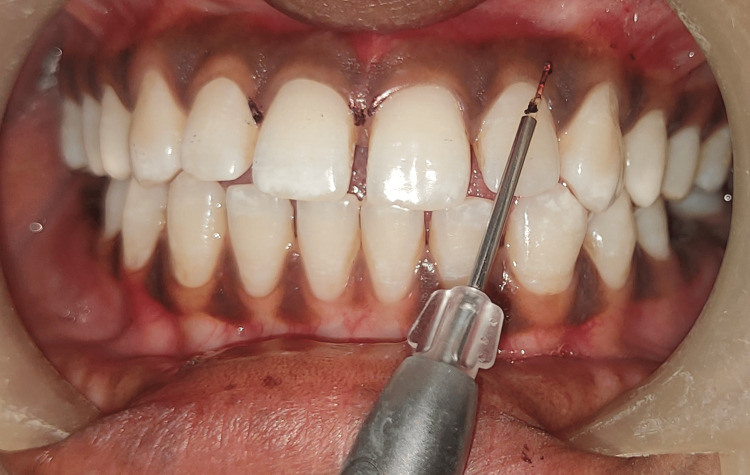
Depigmentation using laser The image shows depigmentation of the upper left anterior quadrant using a diode laser

Laser depigmentation was applied on the left counterpart, as shown in Figure 3.

**Figure 4:**
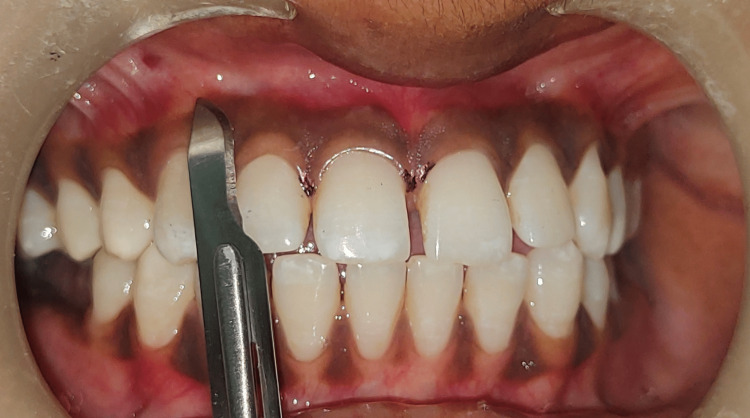
Depigmentation using scalpel The image shows depigmentation of the right anterior upper quadrant using a scalpel

The conventional/traditional method was performed for depigmentation of the maxillary right anterior area from the central incisor to the canine (anterior aesthetic section), using a #15 blade as shown in Figure 4.

**Figure 5:**
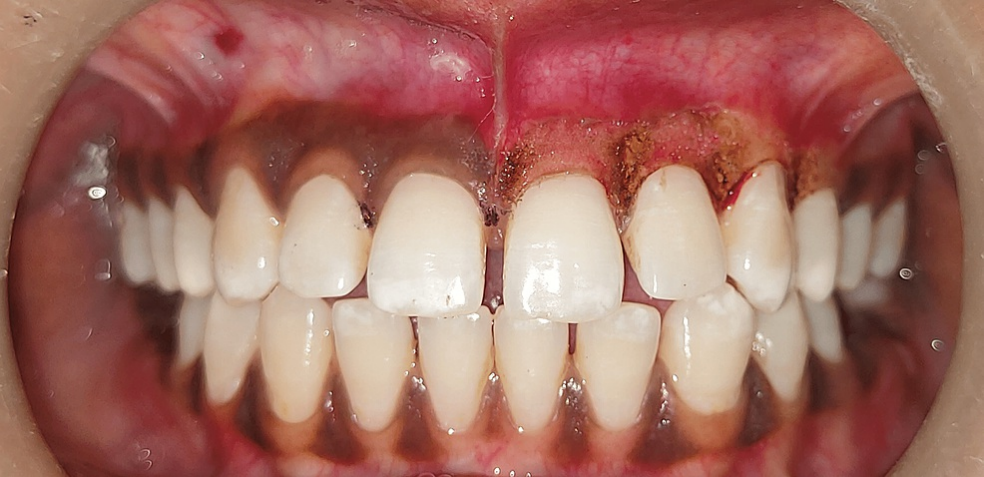
Figure depicting treated site using laser The image shows the left anterior upper quadrant treated using a laser

Figure 5 depicts the treated site using a laser.

**Figure 6:**
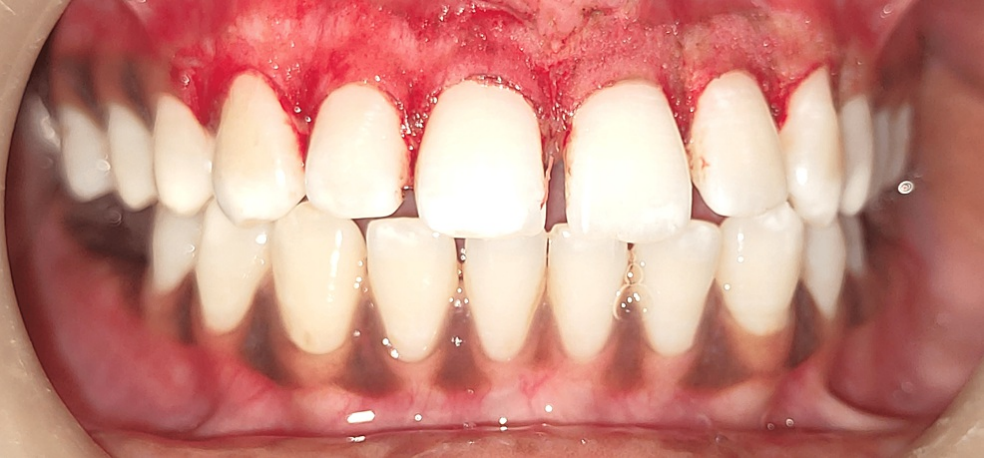
Figure depicting sites treated by scalpel and laser The image shows sites treated by scalpel (right side) and laser (left side)

Figure 6 shows treated areas by scalpel and laser.

**Figure 7:**
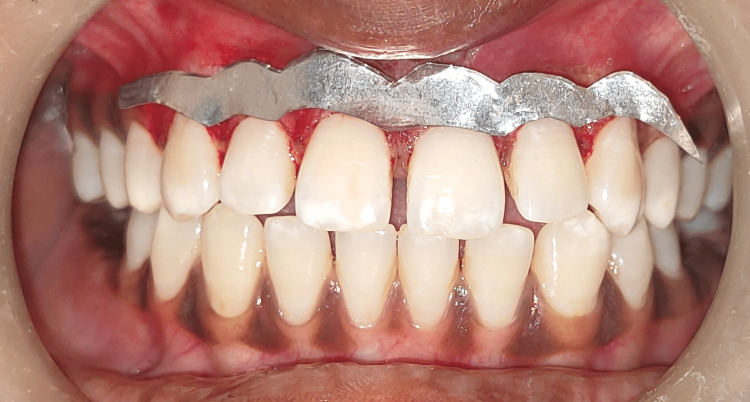
Figure depicting the stent in place The image shows treated sites assessed using a tin foil stent covering the denuded surface

During the surgery, there was no bleeding. Afterward, the corrected area was assessed using a stent, as shown in Figure 7.

**Figure 8:**
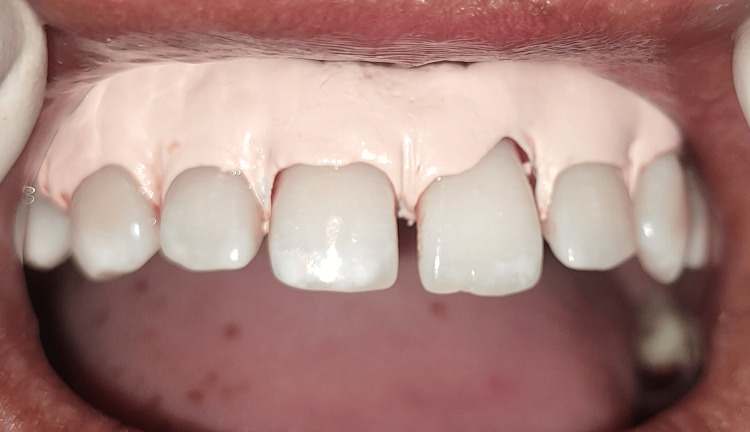
Periodontal pack applied The image shows a periodontal pack applied to cover the treated sites for uneventful healing

 A periodontal pack was applied, as shown in Figure 8.

Following the surgery, the patient was given postoperative instructions, and a nonsteroidal anti-inflammatory drug, diclofenac sodium, was prescribed two times a day for three days. After one week, the patient was called back for reevaluation. Both sides of the wound had healed evenly. The patient had experienced discomfort in the scalpel-treated area for three days after surgery. After a week, the periodontal pack was removed, and the surgical site was examined. The recovery was uneventful one month after surgery, with no postoperative problems. The gingiva was pink, firm, and healthy and had a distinctive look. The patient was pleased with the aesthetic result. The mandibular anterior area was pigmented, but it was not an aesthetic concern for the patient.

## Discussion

Oral pigmentation affects people of all ethnicities. It does not differ much between females and males. On the other hand, the oral epithelium's pigmentation varies in strength and distribution. It varies not just across races but also among people of the same race and within various parts of an individual's oral cavity. Although the degree of pigmentation is also regulated by mechanical, chemical, and physical factors, physiologic pigmentation is most likely determined by heredity [4,7].

One of the first and most popular therapies for this condition was the surgical elimination of unwanted pigmentation. Surgical stripping and split thickness epithelial excision are other terms for the procedure. The technique involves surgically eliminating the gingival epithelium and a thin connective tissue layer beneath it. The stripped connective tissue is then healed with secondary intention. The newly formed epithelium is free from melanin color [6]. An attempt was made to eliminate gingival pigmentation on the patient's maxillary right quadrant. The scalpel approach of depigmentation yielded promising results in this case, both clinically and in terms of patient satisfaction. Scalpel surgery, on the other hand, leads to excessive bleeding during and after the procedure, necessitating the application of a periodontal pack for 7-10 days. The region had entirely improved in 10 days with a normal gingival appearance. Murthy et al. examined three techniques - bur abrasions, surgical blade, and diode laser - on three patients in a case series [8]. Based on their findings, the blade group healed the fastest, possibly related to any thermal damage brought on by the diode laser's coagulation action. We discovered that the scalpel approach was straightforward, adaptable, requiring little time and effort, and affordable [9]. Compared to other surgical techniques, this procedure has a quicker healing rate. Although the surgical removal of the gingiva is less expensive and has a lower likelihood of recurrence, it is painful, uncomfortable after the procedure, causes intra- and postoperative bleeding, and necessitates periodontal dressing. This procedure should not be used if patients have a thinner gingival biotype or narrow papillary regions.

Laser therapy is the most effective method for treating gingival hyperpigmentation [10]. Carbon dioxide (CO_2_, 10,600 nm), neodymium-doped yttrium aluminum garnet (Nd: YAG, 1,064 nm), and diode (980 nm) lasers are the most commonly utilized lasers for gingival depigmentation. Surgical sites may be seen clearly with lasers; moreover, this procedure is associated with fewer postoperative problems such as discomfort, bleeding, edema, infection, and slower wound healing [11]. It is a reliable and secure treatment option with easy access to the interdental papilla and a low recurrence rate. Simple access to the interdental papilla and a low recurrence rate make it a reliable and secure treatment option [12]. Lasers can produce superior aesthetic effects, but they are an expensive approach that requires specialized equipment and takes a lot of space. The laser site reportedly had minor discomfort after surgery compared to the scalpel group. The fact that the protein coagulum produced on the wound's surface acts as a physical wound dressing [13] and plugs the terminals of sensory neurons supports this theory [2]. Using Er: YAG lasers have also been associated with decreased discomfort, according to studies by Azzeh and Kishore et al. Er, Cr: YSGG has been utilized in case reports to remove gingival melanin pigmentation [14]. Additionally, they noted that this laser was excellent, hardly causing minor bleeding or pain. This is in line with previous research that reports that laser therapy offers the benefits of ease of use, quick treatment times, hemostasis, disinfection, and sterilization and that it does not even require a periodontal dressing [15].

## Conclusions

There was no recurrence with any of the treatments examined, and both the approaches had equivalent results in terms of healing. The scalpel method left a bleeding area that needed to be examined after surgery. The burned layer nevertheless served as a surgical dressing to stop the bleeding. Therefore, the laser treatments did not leave any such bleeding. Lasers need technical skills, even if they should only be used with prudence and have the advantage of producing minor bleeding. In addition, the high cost of the laser system must be considered. As a result, within the confines of this case study, it may be stated that both techniques result in equivalent long-term benefits.
